# In situ forming injectable MSC-loaded GelMA hydrogels combined with PD for vascularized sweat gland regeneration

**DOI:** 10.1186/s40779-023-00456-w

**Published:** 2023-04-25

**Authors:** Enhe Jirigala, Bin Yao, Zhao Li, Yi-Jie Zhang, Chao Zhang, Li-Ting Liang, Fan-Liang Zhang, Xing-Yu Yuan, Xian-Lan Duan, Wei Song, Meng-De Zhang, Yi Kong, Xiao-Bing Fu, Sha Huang

**Affiliations:** 1 Research Center for Tissue Repair and Regeneration, Medical Innovation Research Department, Chinese PLA General Hospital and PLA Medical College, Beijing, 100048 China; 2 Institute of Basic Medical Research, Inner Mongolia Medical University, Hohhot, 010110 China; 3 Academy of Medical Engineering and Translational Medicine, Tianjin University, Tianjin, 300072 China; 4 School of Medicine, Nankai University, Tianjin, 300071 China

**Keywords:** Sweat gland, GelMA, In situ niche, Cell differentiation, Tissue incorporation, Vascularization

Dear Editor,

Three dimensional (3D) bioprinted extracellular matrix (ECM) can be used to provide both biochemical and biophysical cues to direct mesenchymal stem cells (MSCs) differentiation, and then differentiated cells were isolated for implantation in vivo using surgical procedures. However, the reduced cell activity after cell isolation from 3D constructs and low cell retention in injured sites limit its application [1]. Methacrylated gelatin (GelMA) hydrogel has the advantage of fast crosslinking, which could resemble complex architectures of tissue construct in vivo [2]. Here, we adopted a noninvasive bioprinting procedure to imitate the regenerative microenvironment that could simultaneously direct the sweat gland (SG) and vascular differentiation from MSCs and ultimately promote the replacement of glandular tissue in situ (Fig. 1a).

**Fig. 1:**
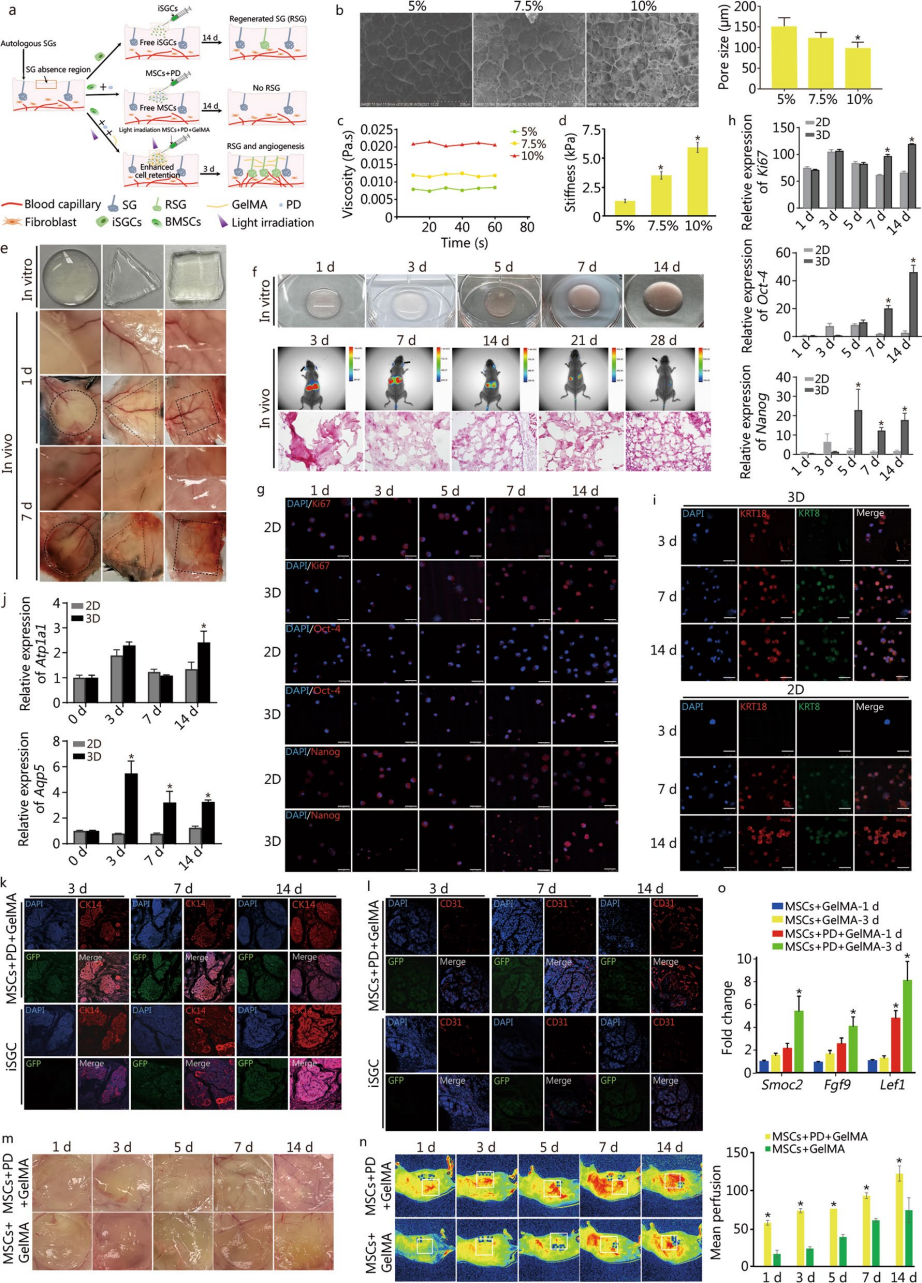
3D bioprinted niche promotes cell differentiation and tissue incorporation. **a** Schematic illustration of the whole process of in vivo transplantation. **b** Scanning electron microscope (SEM) images and pore size of GelMA with 5%, 7.5% and 10% concentrations (Scale bar = 200 μm). **c** Viscosity of GelMA with 5%, 7.5% and 10% concentrations. **d** Stiffness of GelMA with 5%, 7.5% and 10% concentrations (10% vs. 7.5%, 7.5% vs. 5%, ^*^P < 0.05). **e** Shape of bioprinted constructs (circle, triangle, square) in vitro and in vivo after 1 d and 7 d (Scale bar = 500 mm). **f** Degradation of hydrogel in vitro at days 1, 3, 5, 7, and 14, and the degradation of hydrogel and DiI-labeled cell tracing in vivo at days 3, 7, 14, 21, and 28 (Scale bar = 100 μm). **g** Proliferating cells were detected through Ki67 stain and comparison of stemness markers Oct-4 and Nanog between 2D (MSCs + PD) condition and 3D-bioprinted constructs (MSCs + PD + GelMA) at 1, 3, 5, 7 and 14 d of culture (DAPI: blue; scale bar = 50 μm). **h** Transcriptional expression of *Ki67*, *Oct-4* and *Nanog* at days 1, 3, 5, 7, and 14 culture by quantitative real-time polymerase chain reaction (qRT-PCR). Data are mean ± SEM (standard error of mean) (3D vs. 2D, ^*^P < 0.05). **i** Expression of SG-specific markers KRT18 and KRT8 at 3, 7 and 14 d of culture (KRT18: red; KRT8: green; DAPI: blue; scale bar = 50 μm). **j** Transcriptional expression of SG functional marker *Atp1a1* and *Aqp5* in 2D (MSCs + PD) condition and 3D condition (MSCs + PD + GelMA) in days 3, 7, and 14 culture (3D vs. 2D, ^*^P < 0.05). **k** Expression of CK14 and GFP-labeled cells in SG after injection at days 3, 7, and 14 of MSCs + PD + GelMA group and iSGCs group (CK14: red; DAPI: blue; scale bar = 50 μm). **l** Expression of CD31 and GFP-labeled cells in SG after injection at day 3, 7, and 14 of MSCs + PD + GelMA group and iSGCs group (CD31: red; DAPI: blue; scale bar = 50 μm). **m** Macroscopic images of blood vessel formation in vivo after transplantation of MSCs + PD + GelMA group or MSCs + GelMA group at days 1, 3, 5, 7, and 14. **n** Detection and quantification of blood perfusion in vivo after transplantation of MSCs + PD + GelMA group or MSCs + GelMA group at days 1, 3, 5, 7, and 14 (MSCs + PD + GelMA group vs. MSCs + GelMA group, ^*^P < 0.05). **o** Transcriptional expression of vascular induction genes *Smoc2*, *Fgf9* and *Lef1* in days 1 and 3 culture by qRT-PCR (MSCs + PD + GelMA group vs. MSCs + GelMA group, ^*^P < 0.05). SG sweat gland, RSG regenerated sweat gland, BMSC bone marrow mesenchymal stem cell, MSCs mesenchymal stem cells, PD plantar dermis, iSGCs induced sweat gland cells, GelMA methacrylated gelatin

We first investigated the physical characteristics of GelMA hydrogel with different concentrations. Scanning electron microscope (SEM) images revealed that GelMA possessed a highly porous structure and the pore size of GelMA decreased with increasing concentration (Fig. 1b). Rheological testing showed that the viscosity of the GelMA didn’t show significant change with increasing time at a shear rate of 10 rad/s, and the viscosity were gradually increased with the GelMA concentration. The Young’s modulus of GelMA bioinks ranged from 1.1 kPa (5% GelMA) to 5.6 kPa (10% GelMA) (Fig. 1c, d). According to a previous study, uniform pores with around 125 μm could maintain the stemness of MSCs [3]. Without the sacrifice of suitable printibility, 7.5% GelMA was chosen for the following test. Three bioprinted microconstructs were fabricated and the integrity of bioprinted constructs could be successfully maintained in vitro and in vivo (Fig. 1e). In vivo degradation assay showed that the distribution of DiI-labeled cells was extensive and hematoxylin-eosin staining showed few bioink debris and infiltration of cells (Fig. 1f). Low inflammatory response indicating the good histocompatibility of the hydrogel, which is suitable for clinical noninvasive treatment (Additional file 1: Fig. S1).

For further investigation of the biological functions of the GelMA bioink, cell proliferation and differentiation of MSCs encapsulated in the bioink were measured under the regeneration microenvironment of SG in vitro. In our previous study, MSCs could differentiate into SG-like cells in 3D bioprinted construct with SG specific ECM-plantar dermis (PD) [1]. Therefore, PD was introduced into GelMA to direct SG cell fate in vitro. After the identification of MSCs by differentiation experiment (Additional file 1: Fig. S2), we confirmed that PD retains mainly extracellular components of the specific microenvironment, excluding the influence of pre-existing SG cells. The DNA concentration of PD, which laterally reflects the cellular content, was reduced by 90%, while the ECM contents such as collagen and GAGs were well preserved (Additional file 1: Fig. S3). When MSCs were added into the PD containing GelMA, the expression of *Ki67*, *Oct-4* and *Nanog* in the 3D (MSCs + PD + GelMA) construct was increased with culture while decreased in the 2D (MSCs + PD) condition with culture at both the protein and gene level (Fig. 1g, h). For in vitro differentiation, the expression level of SG markers KRT18 and KRT8 elevated at day 3 in 3D group increased with culture (Fig. 1i). The expression of functional sweating marker *Atp1a1* for ion transport and *Aqp5* for water transport in 3D group was higher than those of MSCs + PD culture in 2D condition (Fig. 1j). These results fully demonstrated the excellent role of GelMA in promoting the proliferation and directed differentiation of MSCs.

Next, GelMA-based noninvasive in vivo 3D bioprinting was performed. To better trace the injected cells, we used Green fluorescent protein (GFP)-labeled cells MSCs here. In the MSCs + PD + GelMA group, chimerism was shown in SG tissue 3 d after injection and GFP-labeled cells were increased with time, while traditional induced SG cells (iSGCs-MSC + PD) in our previous study [1] were incorporated into SG tissue until 7 d after injection (Fig. 1k). There was no chimerism observed in MSCs + PD group (Additional file 1: Fig. S4). GFP-labeled cells also showed the expression of SG specific marker KRT18, which demonstrated that MSCs could differentiate into SG cells in vivo (Additional file 1: Fig. S5). Taken the positive role of vascular networks on tissue development and regeneration into account, we further measured the expression of CD31 in the chimeric sites. Interestingly, the expression of CD31 was higher in the MSCs + PD + GelMA group than the iSGCs group in vivo (Fig. 1l), which may indicate the vascular-promoting effects of PD besides its differentiation-inducing effect on MSCs.

In order to figure out the potential role of PD for angiogenesis, we further investigated whether MSC-loaded GelMA combined with PD or not is responsible for the new formation of the blood vessels in vivo and in vitro. Blood vessel formation assay showed that there were increased numbers of vessels that migrated the gels with PD than gels without PD in vivo (Fig. 1m). Analysis using laser speckle imaging revealed increased perfusion in the skin over the gels of PD (Fig. 1n). And the expression of vascular genes significantly increased in the MSCs + PD + GelMA group compared with the MSCs + GelMA group in vitro (Fig. 1o).

In summary, a straightforward and efficient in situ therapeutic strategy was developed to fabricate light-patterning hydrogels which could meet the requirements for biocompatibility, as well as physical, and biochemical features by modifying the porosity and modulus. This strategy was not only to form different patterns but also to induce cell differentiation and promote the iSGCs incorporated into SG tissues through vascular niche and angiogenic properties.

## Supplementary Information

**Additional file 1**. Materials and Methods. **Fig. S1** Histocompatibility of the hydrogel. **Fig. S2** Differentiation capability of BMSCs. **Fig. S3** DNA contents, collagen and GAGs of native tissues and plantar dermis (PD). **Fig. S4** Expression of CK14 and GFP-labeled cells in SG after injection at days 3, 7, and 14 of MSCs PD group. **Fig. S5** MSCs to differentiate into the SG in vivo.

## Data Availability

The data and materials used in the current study are all available from the corresponding author upon reasonable request.

## Abbreviations

ECM: Extracellular matrix
GelMA: Methacrylated gelatin
iSGCs: Induced sweat gland cells
MSCs: Mesenchymal stem cells
PD: Planter dermis
GFP: Green fluorescent protein
SG: Sweat gland
SEM: Scanning electron microscope
