# Supplementary material for: In situ forming injectable MSC-loaded GelMA hydrogels combined with PD for vascularized sweat gland regeneration
Source: Mil Med Res. 2023 Apr 25;10:17. doi: 10.1186/s40779-023-00456-w (PMC10127362; doi:10.1186/s40779-023-00456-w)
Supplement: Supplementary file 1 — Additional file 1. Materials and Methods. Fig. S1 Histocompatibility of the hydrogel. Fig. S2 Differentiation capability of BMSCs. Fig. S3 DNA contents, collagen and GAGs of native tissues and plantar dermis (PD). Fig. S4 Expression of CK14 and GFP-labeled cells in SG after injection at days 3, 7, and 14 of MSCs PD group. Fig. S5 MSCs to differentiate into the SG in vivo. [file 40779_2023_456_MOESM1_ESM.pdf]

Materials and Methods

All animal procedures were approved by the Institutional Animal Care and Use Committee of Chinese PLA General Hospital (Beijing, China). All experiments were repeated independently three times.

Cell isolation and culture

C57BL/6 and C57BL/6-Tg (ACTB-EGFP) 1osb/J(EGFP) (Jackson Laboratory) mice were used for the isolation of MSCs. The procedure followed a published protocol [18]. Briefly, mice aged 7 d old regardless of the gender were sacrificed and immersed in betadine for 15 min. Bilateral femurs and tibias were removed without skin and muscle tissue. Bones were placed in complete MesenCultTM Expansion Medium (Mouse) and crushed with hemostatic forceps. The medium containing bone marrow was then removed and filtered through a 70 μm cell strainer (BD Falcon). Isolated cells were plated in 100 mm dishes and media were changed after 48 – 72 h. The cells were cultured for three passages before use.

Extraction of extracellular matrix

Mice dermal homogenates were extracted from 5-day wild-type C57BL/6 mice (Sibeifu Co., Beijing), the dermis was collected and homogenized with PBS (pH 7.4) in an ice bath. The supernatant was centrifugated at 4 °C for 15 min at 12,000 g. The DNA content was determined using Hoechst 33258 assay (Beyotime, Beijing). The remained DNA was measured to assess the amount of fluorescence with ECM and native tissue using a spectrophotometer (Thermo Scientific, Evolution 260 Bio, USA).

Prepare of GelMA

GelMA derived from porcine gelatin was purchased from EFL company. The light initiator LAP was dissolved in PBS at the concentration of 0.25% in 60 °C water bath for 30 min, then dissolved GelMA in LAP solution in 50 °C water bath for 30min and placed in 37 °C incubator overnight. MSCs were collected from 100-mm dishes, dispersed into single cells, and 100 μl of cell suspension was gently mixed with matrix material under room temperature with cell density 1 million/ml. PD was then gently mixed with GelMA at a ratio of 1:200 and GelMA based hydrogel was patterned under 450 nm UV light.

Scanning electron microscopy

To test the internal porosity, different concentrations of GelMA hydrogels were crosslinked with 450 nm UV laser exposing for 1 min, freeze-dried overnight, and then immersed in liquid nitrogen for 60 s. Then GelMA hydrogels were coated with gold (20 nm) with an Edwards sputter coater and kept desiccated until analysis. Scanning electron microscopy (SEM) was performed using a Hitachi S-4800 scanning electron microscope (Hitachi, Japan).

Rheological testing

A rheological assay of the GelMA hydrogels was carried out on a TA-DHR-3 rheometer (ARES G2). GelMA hydrogels were used on the sample table, and the viscosity was tested with a shear rate within the range of 2 – 100/s at 15 °C. The entire testing process was conducted with aluminum plates with a diameter of 40 mm, and the sample table was positioned 1 mm away from the plate.

Gel modulus measurement

The compression modulus of the GelMA hydrogels was determined by compression test on the universal material testing machine (Instron Model 5567) with a 100 N load at the speed of 1 mm/min until the specimen was broken. Then we took the first 10% curve as a linear relationship. The equation was used to calculate Young's modulus (E represents Young's modulus, σ for stress, and ε for strain). $E = \sigma/\varepsilon$.

Degradation test

The GelMA hydrogels was cultured in complete DMEM medium in 37 °C CO_2 incubator, the medium was changed every 3 day and the morphology of hydrogel was recorded.

HE staining

Tissues were fixed in 4% paraformaldehyde overnight and embedded in Optimal Cutting Temperature (4583, Sakura). Sections were stained with HE staining according to routine histological protocols. Briefly, tissue sections were dewaxed, hematoxylin stained for 8 min, alcohol hydrochloric acid and ammonium hydroxide treated, eosin stained for 5 min, then sealed with resin.

In vitro bioprinting assay

100 μl of MSCs suspension was gently mixed with 7.5% GelMA solution under room temperature

with cell density 1 million/ml. PD or PBS was then gently mixed with GelMA at a ratio of 1: 200 and GelMA based hydrogel was patterned under 450 nm UV light. Cells were divided into two groups: MSCs + PD group (2D), and MSCs + PD + GelMA group (3D).

In vivo bioprinting assay

BALB/cA-nu female mice aged 4 weeks (Sibeifu Co. China) were anesthetized with pentobarbital (100 mg/kg) and received preoperative subcutaneous buprenorphine (0.1 mg/kg). Mice were divided into three groups: iSGC group, MSCs + PD group (2D) and MSCs + PD + GelMA group (3D). Three mice were used in each group. The animals were positioned prone to provide better oxygenation. After scrubbing with betadine, the hydrogel was injected subcutaneously in the dorsal or footpad following light exposure, and all procedure was conducted in a sterile condition. All animals were followed up for 14 d post-engraftment. Mice in each group were sacrificed and hydrogel with skin was sampled on day 14 for histological analysis.

Bioluminescence imaging

MSCs were stained using DiI solution (C1036, Beyotime) and then the labeled cells were mixed with hydrogel and subcutaneously injected. To trace the DiI-labeled cells in vivo, BALB/cA-nu mice were anesthetized and fixed on the working platform and exposed to the light source. Bioluminescence imaging was performed to track the retention and the viability of cells on days 3, 7, 14, 21, and 28 after injection using a Bruker image station system FX PRO (Bruker). Represented images were quantified by detecting the regions of interest (ROIs) with the molecular imaging 7.1.1 software (Bruker).

qRT-PCR

Bioink-encapsulated cells were extracted for RNA isolation by treatment with lyase buffer for 30 min to dissolve the gel. After treatment, samples were centrifuged at 400 g for 10 min to collect the cells. Total RNA was isolated with TRIzol reagent (Invitrogen), and quantity and purity of RNA were detected using NanoDrop 2000c Spectrophotometer (Thermo). RNA was reverse transcribed to cDNA with random primers and RT-qPCR was performed using an RT-qPCR Kit (Takara) on ABI7500 real-time instrument. The data were analyzed using the delta-delta Ct method. Primers were listed below.

**Primers**	**Sequences (5' to 3')**

Nanog-F	CACCCACCCTACTCCATACCAA
Nanog-R	GTGATCCTTCTGTCTCGTCCTT
Oct-4-F	AGGAGATATGCAAATCGGAGACCC
Oct-4-R	CCATAGCCTGGAGCACCAAAGTG
Smoc2-F	CCCAAGCTCCCCTCAGAAG
Smoc2-R	GCCACACACCTGGACACAT
Fgf9-F	ATGGCTCCCTTAGGTGAAGTT
Fgf9-R	TCATTTAGCAACACCGGACTG
Lef1-F	TGTTTATCCCATCACGGGTGG
Lef1-R	CATGGAAGTGTCGCCTGACAG
Atp1a1-F	GACGCCTTTCAGAATGCCTACC
Atp1a1-R	TGTGACCATGATGACCTTAATCCC
Aqp5-F	GCCTTATCCATTGGCTTGTCTGTC
Aqp5-R	CCCAGTCCTCCTCCGGCTCATA
GAPDH-F	CTCTGGAAAGCTGTGGCGTGAT
GAPDH-R	GGAGACAACCTGGTCCTCAGTGTAG

Tissue immunofluorescent staining

Tissues were fixed in 4% paraformaldehyde overnight and embedded in paraffin (Sigma-Aldrich, USA), and sectioned using a slicer (Leica, CM1950, Germany) for 4-μm thickness. Sections were stained with antibodies according to standard immunofluorescence protocols. Briefly, after dewaxed, antigen retrieval with citric acid solution and sodium citrate solution, permeabilized with a solution of 0.1% Triton X-100 (Sigma-Aldrich, USA) in PBS for 5 min and block with 5% goat serum, sections were incubated overnight at 4 °C with the primary antibody below: the antibodies rabbit monoclonal anti-14 (1:300, Abcam), and then immersed with goat anti-rabbit secondary antibody Alexa Fluor® 488 (1:300, ab150077, Abcam) for 2-hour dark incubation at room temperature. Finally, incubated sections were incubated with DAPI Fluoromount-G (0100-20, Southern Biotech) and pictures were taken with a fluorescence microscope (Olympus, BX51) within 24 h.

Blood vessel formation assessment

MSCs suspended with GelMA at a concentration of 2×10^6 cells per ml in a total volume of 200 μl.

The hydrogel was then injected subcutaneously on the dorsal surface of the nu/nu mice (aged 4 weeks). Mice were divided into two groups: MSCs + GelMA group and MSCs + PD + GelMA group. Blood perfusion on the back of the mice was assessed using a custom speckle imaging system on days 1, 3, 5, 7, and 14 after injection of the hydrogel. Laser speckle imaging was quantified by taking the ratio of the perfusion in the region of skin over the implanted hydrogel to the perfusion of the skin.

Statistical analysis

All data were presented as means ± SEM. Statistical analyses were performed using GraphPad Prism9 statistical software (GraphPad, USA). Significant differences were calculated by analysis of variance (ANOVA), followed by the Bonferroni test when performing multiple comparisons between groups. $P < 0.05$ was considered a statistically significant difference.

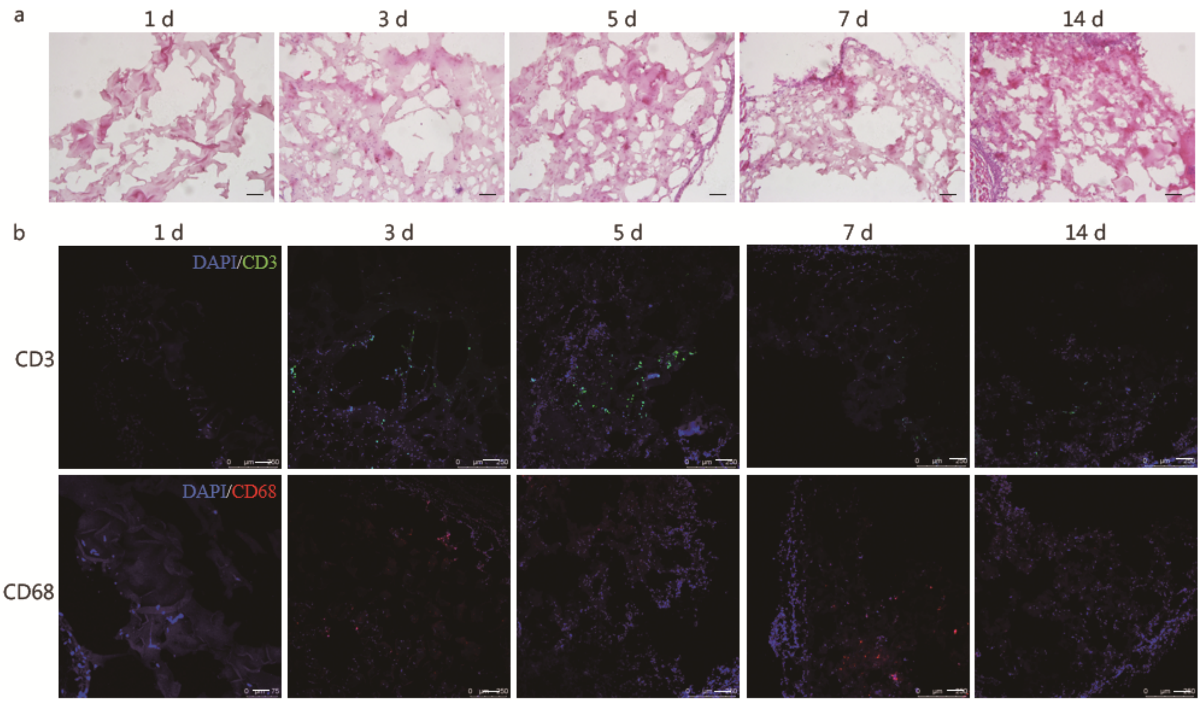

Fig. S1 Histocompatibility of the hydrogel. **a** Macroscopic images of transplanted hydrogel in vivo at days 1, 3, 5, 7, and 14. **b E**xpression of CD3 and CD68 transplanted hydrogel in vivo at days 1, 3, 5, 7, and 14. CD3: specific marker of T cells, CD68: specific marker of macrophage (CD3: green; CD68: red; DAPI: blue; scale bar = 50 μm)

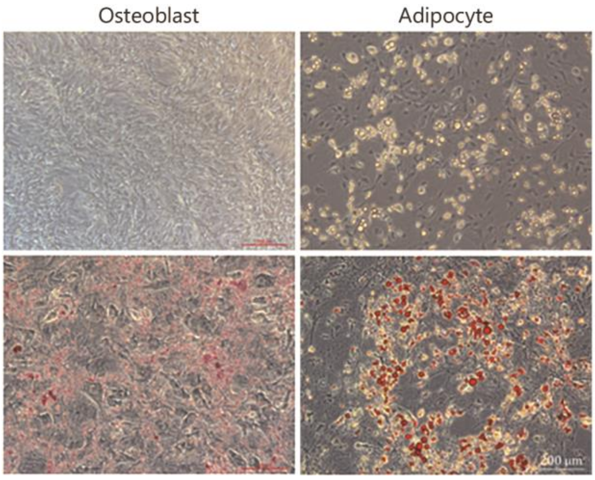

Fig. S2 Differentiation capability of BMSCs. BMSCs differentiate into osteoblasts and adipocytes cultured with differentiated medium. The images of upper row showed cell morphology after induction of osteoblast and adipocyte for 14 d; the images of bottom row showed results of alizarin red staining and oil red O staining. BMSCs bone marrow mesenchymal stem cells

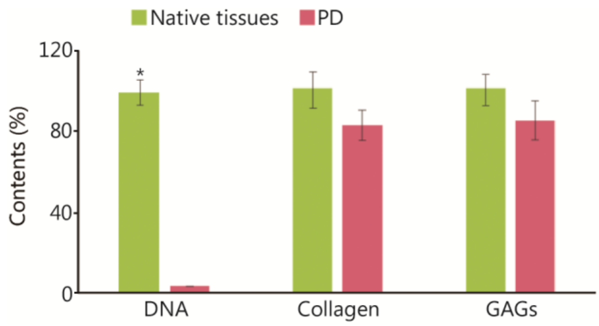

Fig. S3 DNA contents, collagen and GAGs of native tissues and plantar dermis (PD). $^{*}P < 0.05$. GAGs glycosaminoglycans

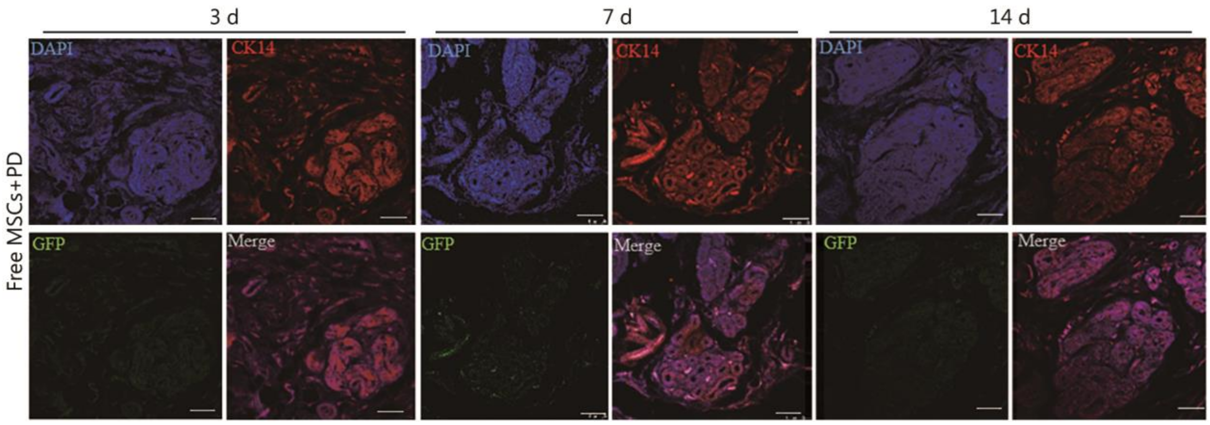

Fig. S4 Expression of CK14 and GFP-labeled cells in SG after injection at days 3, 7, and 14 of MSCs + PD group (CK14: red; DAPI: blue; scale bar = 50 μm). SG sweat gland, MSCs mesenchymal stem cells, PD plantar dermis

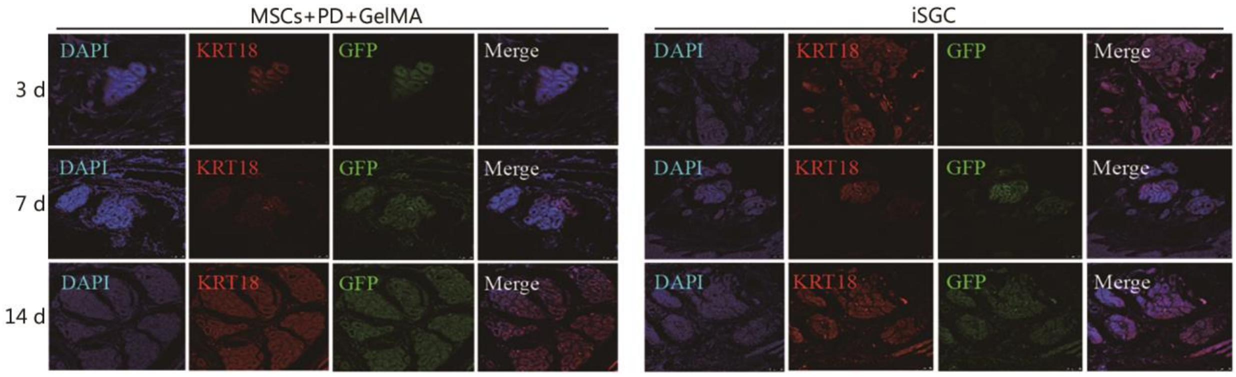

Fig. S5 MSCs to differentiate into the SG in vivo. The expression of KRT18 and GFP-labeled cells in SG after injection at days 3, 7, and 14 of MSCs + PD + GelMA group and iSGC group (KRT18: red; DAPI: blue; scale bar = 50 μm). SG sweat gland, MSCs mesenchymal stem cells, PD plantar dermis, iSGCs induced sweat gland cells, GelMA methacrylated gelatin
